# Effects of Various Drying Parameters on the Volatile and Non-Volatile Compositions of ‘Qiancha 1’ White Tea

**DOI:** 10.3390/foods14213787

**Published:** 2025-11-05

**Authors:** Jinlong Luo, Siyu Liao, Fengjiao Ding, Yuqiao Dai, Zhongying Liu, Ting Yang, Tuo Zhang, Shimao Fang, Yan Li, Lulu Pu, Ke Pan, Wanping Fang, Qiang Shen

**Affiliations:** 1 Guizhou Tea Research Institute, Guizhou Academy of Agricultural Sciences, Guiyang 550006, China9314426840@163.com (K.P.); 2 Tea Refining and Innovation Key Laboratory of Sichuan Province, College of Horticulture, Sichuan Agricultural University, Chengdu 611130, China; 3 College of Horticulture, Nanjing Agricultural University, Nanjing 210095, China

**Keywords:** Qiancha 1, white tea, drying technique, metabolomic, flavor quality

## Abstract

‘Qiancha 1’ is an excellent raw material for manufacturing white tea. The effects of different drying parameters on the quality performance of ‘Qiancha 1’ white tea remain poorly understood, which restricts the precise regulation of the quality of ‘Qiancha 1’ white tea. In this research, we systematically investigated the influence of drying temperature (65 °C, 75 °C, and 90 °C) and drying duration (1 h, 2 h, and 3 h) on its non-volatile and volatile compositions, using sensory evaluation, E-tongue, and non-volatilomic and volatilomic analyses. The results showed that the tea sample dried at 65 °C for 3 h had a sweet, mellow, and fresh flavor and scored 95 points, but high-temperature drying (90 °C) could promote increased bitterness and decreased sweetness. High-temperature drying was closely related to a caramel-like and milk-like flavor, which promoted an increase in the content of terpenoids, heterocycle compounds, and esters. During drying, the flavonoid and phenolic acid content increased markedly, contributing to bitterness and astringency, while nucleotides, amino acids, and their derivatives decreased, leading to a reduced umami intensity. A total of 37 key taste-active metabolites were identified, including bitter compounds (e.g., alkaloids), sweet compounds (e.g., phenolic acids), and umami compounds (e.g., nucleotides), whose dynamic changes directly influenced the taste profile of white tea. High-temperature drying promoted an increase in the content of volatile metabolites, such as terpenoids, heterocyclics, and esters, while low-temperature and long-duration drying was beneficial for preserving volatile metabolites like heptanal. 2-Methoxy-3-(1-methylethyl)-pyrazine was determined as the volatile compound with the highest rOAV, providing a sweetness and caramel-like flavor. Overall, the metabolomic analysis revealed that the content of flavonoids and phenolic acids increased after the drying process, which was related to the bitter and astringent taste of the tea liquor. The content of nucleotides, amino acids, and their derivatives decreased after drying, which caused the umami of the tea liquor to weaken. This study provides a theoretical basis for the optimization of the ‘Qiancha 1’ white tea drying process.

## 1. Introduction

‘Qiancha 1’ (*Camellia sinensis*) is a tea cultivar independently developed in Guizhou Province, China. It was selected from Mei-Tan-Tai-cha populations (registration number: GPD Tea plant (2019) 520007, Available online: http://202.127.42.47:6010/index.aspx (accessed in 12 April 2019)), which is suitable for processing into green tea, white tea, and black tea, yielding products with excellent flavor quality [1,2,3]. The white tea made from ‘Qiancha 1’ is rich in aldehydes and ketones, which is a characteristic of high-quality white tea [4].

White tea is one of six major tea types in China and is popular among consumers for its fresh, sweet flavor and associated health function [5,6]. Withering and drying are key procedures in white tea processing, which have an important effect on the flavor of white tea [7]. Drying, the final process in white tea processing, plays a vital role in quality formation in the thermochemical reaction, causing a flavor boost [8,9]. Research shows that drying temperature and time are critical factors in determining the flavor of white tea [9].

Hot-air drying is a commonly used drying technology in tea production. Drying parameters, primarily temperature and duration, are key factors that influence the final flavor quality of tea [9,10,11]. The primary objective of the drying process is to reduce the moisture content of tea leaves to a level suitable for safe storage, thereby preventing microbial spoilage and undesirable chemical degradation [12]. Concurrently, another aim of drying is to promote and modulate the complex physicochemical transformations within the leaves. The freshness and initial treatment of leaves are closely related to the quality performance of white tea and are mainly affected by plucking and sorting. Plucking standards determine the grade and category of white tea, while sorting determines its purity [9]. White tea processing involves only two main steps: withering and drying. The drying stage directly determines the sensory characteristics of the final product, as well as the composition and concentration of its aroma and taste compounds. It induces the Maillard reaction, which contributes sweet and lightly baked notes [13]. Furthermore, drying promotes the isomerization and oxidation of certain constituents, resulting in the mellow and sweet taste profile characteristic of high-quality white tea. Studies have shown that drying temperature is strongly correlated with the content of flavonoid compounds in white tea [1,9]. Furthermore, drying temperature significantly influences the composition of aromatic compounds, particularly esters, alcohols, aldehydes, and monoterpenes [8,9]. As drying time increases, the flavor profile of white tea undergoes a noticeable transition, evolving from fresh and green to floral, fruity, sweet, woody, and eventually bitter notes [14]. In summary, the drying process profoundly shapes the flavor quality of white tea. However, there remains a lack of comprehensive research on the combined effects of drying temperature and duration on its flavor attributes. Current studies on the influence of drying parameters on white tea quality also suffer from insufficient systematic investigation.

As a traditional method for assessing tea quality, sensory evaluation offers an intuitive reflection of tea characteristics, though its outcomes might be influenced by subjectivity [15]. In recent years, the rapid development of modern analytical techniques has provided new approaches and methodologies for the precise evaluation and systematic investigation of tea flavor through the combined application of methods, such as sensory evaluation, E-tongue (electronic tongue), and metabolomics. The E-tongue, a sensor technology that simulates the human taste system, enables objective and rapid detection of taste compounds in tea. It compensates for the limitations of sensory evaluation. Metabolomics, on the other hand, allows for the comprehensive analysis of changes in metabolic profiles within tea leaves, unveiling the mechanistic basis of how different drying processes shape the flavor profile of ‘Qiancha 1’ white tea [16]. However, the combined effects of different combinations of drying parameters (temperature and duration) on quality performance are not clear. This study utilized fresh leaves of ‘Qiancha 1’ at the one-bud and two-leaf stage as a raw material for white tea production. Different drying processes were applied. An integrated approach combining sensory evaluation, E-tongue analysis, and widely targeted metabolomics was employed to systematically investigate the effects of various drying techniques on the flavor profile of ‘Qiancha 1’ white tea. This research aimed to clarify the influence of different drying processes on sensory quality and taste under different drying conditions. Moreover, it also identified differential metabolites closely associated with the flavor quality of ‘Qiancha 1’ white tea. This study provided a theoretical basis and technical support for optimizing the drying process of ‘Qiancha 1’ white tea and improving its overall quality while also serving as a reference for research on the processing suitability of the ‘Qiancha 1’ cultivar.

## 2. Materials and Methods

### 2.1. Chemicals

Acetonitrile, methanol, and hexane were purchased from Merck KGaA (Darmstadt, Germany). Formic acid was purchased from Aladdin (Shanghai, China). NaCl, KCl, tartaric acid, ethanol, KOH, HCl, and AgCl were purchased from Sinopharm Chemical Reagent Co., Ltd. (Shanghai, China). A special solution for GL1 preconditioning was purchased from Yingsheng Hengtai Technology (Beijing, China).

### 2.2. Tea Sample Preparation

**Figure 1:**
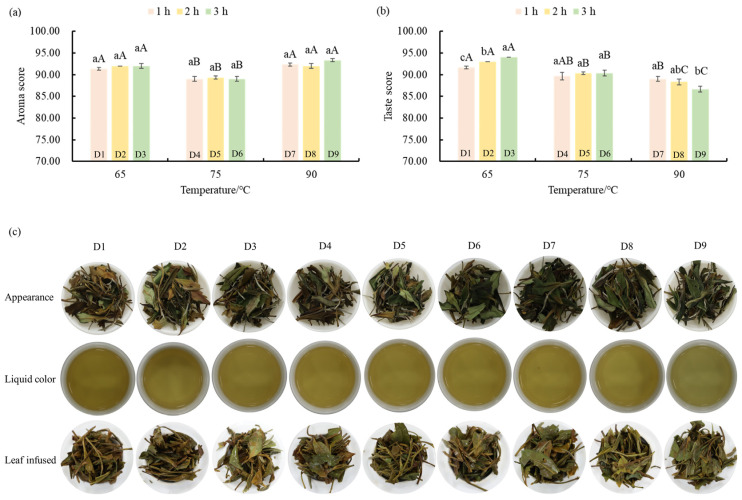
E-tongue detection results of ‘Qiancha 1’ white tea. (**a**) Aroma rating bar chart, (**b**) tasting rating bar chart, and (**c**) the appearance of tea liquor and infused leaves of samples treated by different drying temperatures and times. Note: The values are the mean of three repeated sensory ratings, and the error bars represent the standard error (*n* = 3). Lowercase letters represent the multiple comparison results of different drying times treated at the same temperature at the 0.05 level. Capital letters represent the multiple comparison results of sensory evaluation scores of the tea samples. The same letter indicates no significant difference between the samples. The numbers and corresponding treatment conditions are as follows: D1 (65 °C drying for 1 h), D2 (65 °C drying for 2 h), D3 (65 °C drying for 3 h), D4 (75 °C drying for 1 h), D5 (75 °C drying for 2 h), D6 (75 °C drying for 3 h), D7 (90 °C drying for 1 h), D8 (90 °C drying for 2 h), and D9 (90 °C drying for 3 h).

The fresh tea leaves were produced at Langzhu Organic Industry Co., Ltd., in Zunyi City, Guizhou Province, China. The standard for the leaves was on the bud and two leaves. Withering was performed at an average temperature of 25 °C for 36 h. Then, the withered leaves were treated with different drying parameters to obtain tea samples. The drying parameters were based on previous researchers’ reports and changed slightly according to the actual situation [8,9,17]. All drying processes were conducted in a CC-6HS-9B tea baking machine (Fujian Jiayou Machinery Technology Co., Ltd., Quanzhou, China). The specific processing parameters for these tea samples were D1 (65 °C drying for 1 h), D2 (65 °C drying for 2 h), D3 (65 °C drying for 3 h), D4 (75 °C drying for 1 h), D5 (75 °C drying for 2 h), D6 (75 °C drying for 3 h), D7 (90 °C drying for 1 h), D8 (90 °C drying for 2 h), and D9 (90 °C drying for 3 h) (Figure 1). A blank control (CK) sample was obtained by freezing the withered leaves with liquid nitrogen fixation and then freeze-drying by a vacuum freeze-drying machine (Scientz-100F, Xinzhi Technology, Ningbo, China). Each tea sample contained three replicates. These samples were spread at room temperature immediately after drying to cool the leaves and then packed in polyethylene bags. All the samples were stored at −80 °C.

### 2.3. Sensory Evaluation

The sensory evaluation of the white tea samples (D1~D9 and CK tea samples) was conducted according to the method reported by Hua [18]. All taste substances were food-grade to ensure safety. No human ethics committee or formal documentation process is available, but appropriate protocols for protecting the rights and privacy of all the participants were utilized during the execution of this research, e.g., no coercion to participate, full disclosure of the study requirements and risks, written or verbal consent of the participants, no release of participant data without their knowledge, and ability to withdraw from this study at any time. Five trained tea judges (two males and three females, aged 20~40 years) from the Guizhou Tea Research Institute participated in the assessment. The evaluations were performed in a clean, dry room maintained at 26 ± 1 °C.

The specific operation was as follows: A 3.0 g tea sample was put into a 150 mL tasting cup, filled with freshly boiled water, and covered with a lid. After infusing for 5 min, the tea liquor was quickly filtered. The tea judges evaluated and recorded the quality factors of the tea, including appearance, brewing color, aroma, taste, and brewing leaves. Each quality factor was scored by 100 points. Finally, the total sensory quality score of the white tea samples was obtained according to appearance—25%, soup color—10%, aroma—25%, taste—30%, and leaf bottom—10%.

### 2.4. E-Tongue Evaluation

To prepare for E-tongue analysis, 3.0 g of tea powder was soaked in 150 mL of boiling water for 5 min and then filtered to obtain the tea liquor. Then, 35 mL of the tea liquor was poured into a 50 mL dedicated cup, and data were collected alternately with cleaning. Each tea sample contained four replicates. The specific operational details and data processing methods referred to the method by Ting Yang [15]. For each sample, four replicates of ‘bitter’, ‘astringent’, and ‘umami’ and five replicates of ‘sweet’ were performed for each sample, and the data of the last three replicates were recorded for analysis. The E-tongue data were calculated using the following formula:

V = Vs. − Vr

where V is the relative potential value of the sample solution (used to evaluate the basic value of taste); Vs. is the output value of the membrane potential signal of the taste sensor in the sample solution; and Vr is the output value of the membrane potential signal of the taste sensor in the reference solution.

### 2.5. Non-Volatile Metabolomics Analysis

To obtain tea powder, the tea samples were ground by a mixed grinder (MM400, Retsch, Haan, Germany) at 30 Hz for 90 s. Then, 50 mg of the tea powder was dissolved in 70% methanol, vortexed for 30 s every 30 min for a total of 6 cycles. Then, the mixed liquid was centrifuged at 13,523 g for 3 min and filtered with SCAA-104 (0.22 μm, Anpu Technology, Shanghai, China). The supernatant was used for non-volatile metabolomics analysis. The non-volatile metabolomics analysis method referred to the experimental methods of Dai [1].

### 2.6. Volatile Metabolomics Analysis

#### 2.6.1. HS-SPME Extraction

The method of volatile metabolomics analysis was employed with headspace solid-phase microextraction coupled with gas chromatography–mass spectrometry (HS-SPME-GC-MS) (Agilent, Santa Clara, CA, USA), according to the method reported by Fengjiao Ding [19]. Tea samples were ground with liquid nitrogen and vortexed to obtain a tea sample powder. Then, 500 mg of the powder, a saturated NaCl solution, and 10 μL (50 μg/mL) internal standard (3-Hexanone-2,2,4,4-d4, CAS: 24588-54-3) were transferred into a 20 mL headspace vial. The vial was equilibrated at 60 °C for 5 min, and a 120 μm DVB/CAR/PDMS fiber (Agilent, USA) was exposed to the headspace at 60 °C for 15 min. The fiber was conditioned at 250 °C for 5 min before sampling.

#### 2.6.2. GC-MS Analysis

The fiber was desorbed at the injection port at 250 °C for 5 min. GC-MS analysis was carried out using an Agilent 7890B GC (Agilent, USA) coupled to a 7000D Agilent mass spectrometer (Agilent, USA) equipped with a 30 m × 0.25 mm ×0.25 μm, DB-5MS (Agilent, USA) capillary column. High-purity helium (>99.999%) was used as the carrier gas at a constant flow of 1.2 mL/min. The temperature programs were as follows: the initial oven temperature was 40 °C for 3.5 min; then, it was raised to 100 °C at 10 °C/min, increased to 180 °C at 7 °C/min, and finally ramped to 280 °C at 25 °C/min and kept for 5 min. Mass spectra were conducted in electron impact (EI) ionization mode at 70 eV. The quadrupole mass detector, ion source, and transfer line temperatures were set at 150 °C, 230 °C, and 280 °C, respectively.

#### 2.6.3. Identification and Quantification of Volatile Compounds

The mass spectrometry data were analyzed using MassHunter software (v12.0). The volatile compounds were identified by matching the spectra against the Metware database. Quantification was performed using internal standard calibration based on internal standard peak areas. This method was also referred to in Ding’s report [19].

#### 2.6.4. Relative Odor Activity Value Analysis

Relative odor activity (rOAV) is a metric used to identify the key volatile compounds in food based on the sensory threshold of volatile compounds, which was used to elucidate the contribution of each compound to the overall aroma characteristics of the tea samples. The calculation formula of rOAV was as follows [19]:

$$\mathrm{rOAV}\;=\;\frac{C}{T}$$

where rOAV refers to the relative odor activity value of the volatile compound, C refers to the relative content of the volatile compound (μg/g), and T refers to the threshold of volatile compounds in water (μg/g).

### 2.7. Statistical Analysis

Microsoft Excel 2021 was used to organize the data and draw a Mirror histogram. Statistical significance was assessed through ANOVA (analysis of variance) with SPSS 21 (IBM Corp, Armonk, NY, USA). OPLS-DA (orthogonal partial least squares discriminant analysis), the ring diagram, heatmap analysis, and vene diagram were performed using Metware Cloud (available online: https://cloud.metware.cn/ (accessed on 20 March 2024)).

## 3. Results and Discussion

### 3.1. Sensory Analysis of White Tea Samples with Different Drying Treatments

In order to determine the difference in sensory quality of ‘Qiancha 1’ white tea with different drying treatments, a sensory evaluation of the samples was carried out, and the results are shown in Table S1. In all the samples, D3 obtained the highest score (91.23 ± 0.26), which showed that this drying process (65 °C drying for 3 h) was beneficial to the sweet, mellow, and fresh quality characteristics (Figure 1a,b). Furthermore, there were significant differences in the color performance of the samples. The color of the dry tea, tea liquor, and infused leaves of D1 to D9 gradually changed from yellow to green (Figure 1c). The reason for this phenomenon was that the water-loss rate of leaves in low-temperature drying was slow, and the oxidation reaction of polyphenols produced oxidation products, such as theaflavins, resulting in a yellow-colored tea. On the contrary, high-temperature drying rapidly inactivated the activity of polyphenol oxidase, avoided the oxidation of tea polyphenols, and maintained the green color of the white tea samples. For aroma characteristics, all the treated tea samples had a sweet aroma. Additionally, the samples (D1, D2, and D3) dried at 65 °C had a fresh aroma, while the samples (D7, D8, and D9) dried at 90 °C had a milk-like and caramel-like aroma. According to the results of the sensory evaluation (Table S1), drying temperature had a significant effect on the aroma performance of ‘Qiancha 1’ white tea, but drying time did not affect its aroma performance (89.00 ± 0.58~93.33 ± 0.33). There was no interaction between drying temperature and time on aroma performance. Regarding the taste performance of ‘Qiancha 1’ white tea, all the tea samples had a sweet and mellow taste. The samples (D1, D2, and D3) dried at 65 °C had a refreshing taste. However, the samples (D7, D8, and D9) dried at 90 °C had a bitter taste (5.21 ± 0.06~6.00 ± 0.03). Similar to the effect of the drying parameters on aroma, drying temperature influenced the taste performance of ‘Qiancha 1’ white tea, but drying time did not affect it. As shown in Table S2, drying temperature and time had an interaction effect on the taste performance of ‘Qiancha 1’ white tea, which presented as different parameter combinations had a differential effect on taste.

In our research, with an increase in drying temperature, the color of dry tea, tea liquor, and infused leaves changed from yellow to green, and the green flavor and fresh taste decreased. This revealed that a low temperature for a long time was conducive to the retention of umami and sweet flavors in ‘Qiancha 1’ white tea, which was similar to previous studies [9,20]. Moreover, the high-temperature (90 °C) dried samples (D7, D8, and D9) had a milk-like flavor, showing that high-temperature drying was conducive to the formation of the milk-like flavor in white tea [21]. Based on the comprehensive sensory evaluation results, we discovered that the effect of drying temperature on the flavor quality of ‘Qiancha 1’ white tea was higher than that of drying time.

### 3.2. Effect of Different Drying Treatments on the Taste Characteristics of ‘Qiancha 1’ White Tea

**Figure 2:**
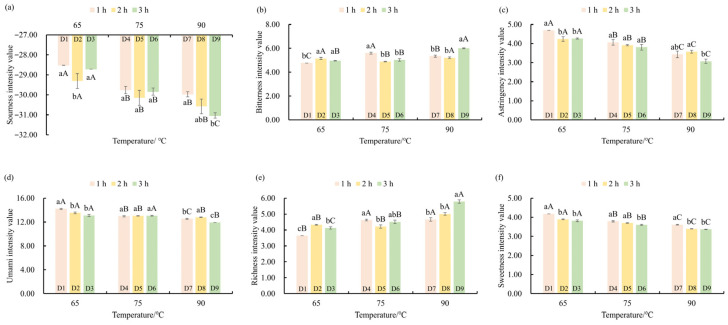
E-tongue analysis of ‘Qiancha 1’ white tea processed under different drying parameters. Histogram showing the intensity values of sourness (**a**), bitterness (**b**), astringency (**c**), umami (**d**), richness (**e**), and sweetness (**f**). Note: The values are the mean of three repeated sensory ratings, and the error bars represent the standard error (*n* = 3). Lowercase letters represent the multiple comparison results of different drying times treated at the same temperature at the 0.05 level. Capital letters represent multiple comparison results of the sensory evaluation scores of the tea samples. The same letter indicates no significant difference between the samples. The numbers and corresponding treatment conditions are as follows: D1 (65 °C drying for 1 h), D2 (65 °C drying for 2 h), D3 (65 °C drying for 3 h), D4 (75 °C drying for 1 h), D5 (75 °C drying for 2 h), D6 (75 °C drying for 3 h), D7 (90 °C drying for 1 h), D8 (90 °C drying for 2 h), and D9 (90 °C drying for 3 h).

In order to objectively evaluate the effect of drying temperature and time on the taste performance of ‘Qiancha 1’ white tea, we employed the E-tongue to detect and analyze its taste quality (Figure 2 and Table S3). We found that drying temperature and time had a significant effect on the taste of white tea, which was sour, bitter, astringent, umami, sweet, and rich. Moreover, in addition to astringency, there was an interaction between drying temperature and drying time on the taste of white tea, which was sour, bitter, umami, sweet, and rich (Table S4). The acidity intensity of ‘Qiancha 1’ white tea decreased with the increase in drying temperature. When the drying temperature was 90 °C, the sourness intensity decreased from −28.52 ± 0.00 to −31.03 ± 0.14 with increased drying time. All the samples had a sour taste of −13, so the sour taste could not be perceived in them. When the drying temperature was 90 °C, the bitterness intensity showed an increasing trend with increased drying time. In all the samples, the white tea dried at 90 °C for 3 h had the highest bitterness intensity.

The intensity of astringency and umami in the ‘Qiancha 1’ white tea samples showed a significant decreasing trend with increasing drying temperature (*p* < 0.05). When the drying temperature was set at 65 °C, the umami intensity decreased progressively with extended drying time. Furthermore, drying temperature was closely correlated with the taste richness of the tea liquor. Under high-temperature drying conditions (90 °C), the taste richness of the tea liquor exhibited a clear increasing trend with a prolonged drying duration. In contrast, the sweetness of the tea liquor decreased with a high drying temperature. Moreover, when the drying temperature remained constant, the sweetness intensity decreased as drying time increased.

In summary, there existed a close relationship between drying temperature and the taste profile of ‘Qiancha 1’ white tea. An increase in drying temperature led to enhanced bitterness and a rich taste of the tea liquor, but it reduced the intensity of umami, sweetness, and astringency [18]. When the drying temperature remained constant, the intensity of umami and sweetness exhibited an inverse relationship with drying time. Given that umami and sweetness were key contributors to the pleasant taste of ‘Qiancha 1’ white tea, our findings confirmed that high-temperature and prolonged drying would result in a reduction in these desirable flavors. Therefore, to preserve the desirable taste quality of ‘Qiancha 1’ white tea, high-temperature and extended drying practices should be avoided in actual production.

### 3.3. Non-Volatile Metabolite Analysis of ‘Qiancha 1’ White Tea

**Figure 3:**
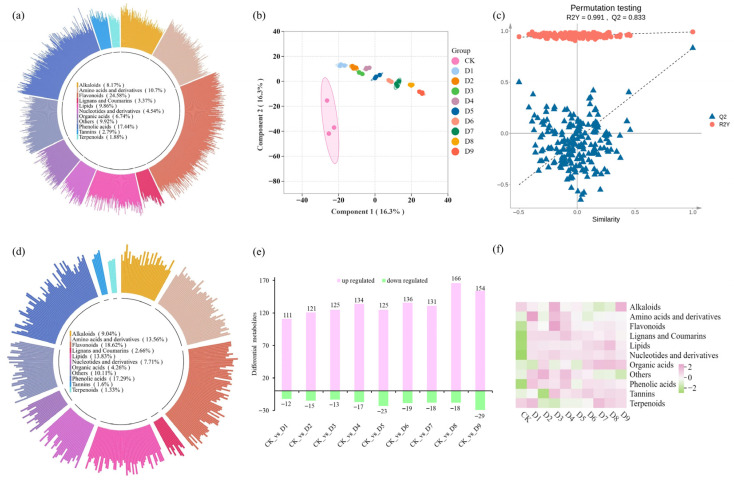
Non-volatile compound composition and multivariate analysis. (**a**) Numbers and percentages for each subcategory of all non-volatile compounds. (**b**) PLS-DA score plot. (**c**) Cross-validation results based on 200 calculations by using a permutation test. (**d**) Number and percentages for each subcategory of differential non-volatile compounds. (**e**) Mirror histogram of differential non-volatile compounds. (**f**) Differential. Note: The numbers and corresponding treatment conditions are as follows: D1 (65 °C drying for 1 h), D2 (65 °C drying for 2 h), D3 (65 °C drying for 3 h), D4 (75 °C drying for 1 h), D5 (75 °C drying for 2 h), D6 (75 °C drying for 3 h), D7 (90 °C drying for 1 h), D8 (90 °C drying for 2 h), and D9 (90 °C drying for 3 h).

Metabolomics analysis was performed on the CK and ‘Qiancha 1’ white tea samples with different drying treatments. A total of 1542 non-volatile compounds were identified (Table S5). They were categorized into 11 classes (Figure 3a), including flavonoids (379, 24.58%), phenolic acids (269, 17.44%), amino acids (165, 10.70%), lipids (152, 9.86%), alkaloids (126, 8.17%), organic acids (104, 6.74%), nucleotides and derivatives (70, 4.54%), lignans and coumarins (52, 3.37%), tannins (43, 2.79%), terpenoids (28, 1.88%), and others (153, 9.92%).

To investigate the effects of different drying treatments on non-volatile metabolites in ‘Qiancha 1’ white tea, orthogonal partial least squares–discriminant analysis (OPLS-DA) was further employed to achieve group separation and calculate the VIP (variable importance in projection) values for all metabolites in the tea samples. The OPLS-DA score plot revealed significant differences among the ten samples, with a clear separation observed between the CK sample and the samples treated by different drying processes (D1–D9) (Figure 3b). This indicated that variations in the content of non-volatile metabolites were the main factor contributing to differences in taste quality among the various white tea samples subjected to different drying processes. The data were suitable for further analysis. The small within-group variation among biological replicates suggested that the observed differences in non-volatile metabolite profiles were reliably attributed to the drying treatments. A permutation test with 200 iterations was performed to validate the OPLS-DA model (Figure 3c). The intercepts of the regression lines for both the *Y*-axis and Q^2^ were below 0, demonstrating that the model was not overfit and the fitting results were statistically acceptable [22].

A total of 376 discrepant non-volatile metabolites were identified in all the tea samples (CK and D1 to D9) (Table S6). They were classified into 11 classes, including flavonoids (70, 18.62%), phenolic acids (65, 17.29%), lipids (52, 13.83%), amino acids and derivatives (51, 13.56%), alkaloids (34, 9,04%), nucleotides and derivatives (29, 7.71%), organic acids (16, 4.26%), lignans and coumarins (10, 2.66%), tannins (6, 1.60%), terpenoids (5, 1.33%), and others (38, 10.11%). Figure 3e shows that the number of up-regulated metabolites was significantly higher than the number of down-regulated metabolites across all comparisons, indicating substantial accumulation of various non-volatile metabolites during the drying process. This suggests that drying methods influence the composition of non-volatile metabolites in ‘Qiancha 1’ white tea. Overall, compared to CK, the number of metabolites with an increased abundance far exceeded those with a decreased abundance. This demonstrates that drying markedly affected most non-volatile metabolites in ‘Qiancha 1’ white tea, and these changes in differential metabolite content were a key factor shaping taste quality.

As shown in Figure 3f, compared to CK, the drying treatments significantly influenced the content of discrepant non-volatile metabolites in ‘Qiancha 1’ white tea, and the flavonoid content in the samples (D1 to D9) increased markedly. Flavonoids were the major contributors to the bitterness and astringency of the tea liquor [23]. This indicates that the drying process considerably affected the flavonoid content and thereby influenced the sensory profile of ‘Qiancha 1’ white tea. Similarly, the total phenolic acid content was significantly higher in D1-D9 than in CK. Most phenolic acids were associated with bitter and astringent tastes [24]. The drying process notably enhanced their accumulation (Figure 3f), which was consistent with previous findings [10]. The majority of phenolic acids exhibited consistent patterns of changes in response to different drying treatments, a trend that aligned with findings reported in previous studies [18]. Lipids, which serve as important precursors for aroma formation, significantly contribute to the development of tea flavor [25]. The content of lipids in the ‘Qiancha 1’ white tea samples (D1 to D9) was significantly higher than that in CK, suggesting that the drying process enhanced the flavor of white tea. Moreover, different drying treatments resulted in distinct variation patterns among individual lipid subclasses. The contents of 9S-hydroxy-10E-12Z-octadecadienoic acid, 13(S)-hydroxyoctadeca-9Z-11E-dienoic acid, alpha-hydroxylinoleic acid, methyl linolenate, dodecanoic acid, and 4-Oxo-9Z,11Z,13E,15E-octadecatetraenoic acid decreased after drying. In contrast, the contents of LysoPC and LysoPE increased after drying. This was consistent with the findings reported in previous studies [26,27]. Except for D2 and D6, the contents of amino acids and their derivatives exhibited a decreasing trend as the drying temperature increased, which was consistent with previous findings [18]. The reason for the decrease in amino acids and their derivatives was that the high temperature induced their conversion into compounds such as pyrazines, indoles, and p-cresol through physical and chemical reactions, including decarboxylation, deamination, and Maillard reactions with carbonyl compounds (e.g., soluble sugars, polyphenols, and ascorbic acid) [11,28]. The content of alkaloids is positively correlated with the perceived bitterness of tea infusions [29]. In the sensory evaluation, the infusion of D9 exhibited a pronounced bitter taste, which was consistent with its high bitterness intensity value measured by E-tongue. This phenomenon was likely attributed to the high alkaloid content in D9. The content of nucleotides and their derivatives exhibited a declining trend with increasing drying temperature and duration (Figure 3f). Since nucleotides and their derivatives are known to contribute to the umami taste of tea infusions [7,30], the umami intensity of ‘Qiancha 1’ white tea was consequently reduced as the drying temperature and time increased. Except for D2 and D5, the content of organic acids in these tea samples followed the order: 90 °C (D7, D8, D9) > 75 °C (D6, D4) > 65 °C (D3, D1). Therefore, it could be inferred that for ‘Qiancha 1’ white tea, the content of organic acid increased with rising drying temperature, specifically demonstrating a trend of 90 °C > 75 °C > 65 °C. Organic acids are associated with the thickness and richness of tea infusions [31]. The E-tongue results indicated that high-temperature drying enhanced the thickness of the ‘Qiancha 1’ white tea infusion. Notably, the contents of some compounds increased after drying, including 2-hydroxyisocaproic acid, benzoylformic acid, succinic acid, aminomalonic acid, and methyl 2-furoate, which suggested a potential positive correlation with the perceived thickness of the infusion. However, this relationship requires further investigation for validation. In contrast, the contents of 2-aminoisobutyric acid*, phosphoenolpyruvate, ethyl 3-hydroxybutyrate, and iminodiacetic acid* decreased markedly after the drying process. Except for D1, the terpenoid content across all the tea samples exhibited a consistent pattern: 90 °C (D7, D8, D9) > 75 °C (D6, D5, D4) > 65 °C (D2, D3). Terpenoids are known to contribute to the aroma profile of tea and represent key compounds responsible for its fragrance [32]. Consequently, a high drying temperature appeared to enhance the aroma of ‘Qiancha 1’ white tea. Lignans and coumarins, which belong to phenylpropanoids, are associated with astringent sensory attributes [7]. Except for D4, their content followed the order: 65 °C (D1, D2, D3) > 75 °C (D5, D6) >90 °C (D7, D8, D9). This result was consistent with the E-tongue findings, which indicated imperceptible astringency in the tea samples dried at high temperatures. Thus, an increased drying temperature likely contributed to a reduction in the contents of lignan and coumarin, thereby mitigating astringency in ‘Qiancha 1’ white tea. Among tannins, the contents of 3,3’-O-dimethylellagic acid, proanthocyanidins, and theaflagallin increased after drying, whereas the contents of arecatannin C1 and procyanidin C2 decreased. In summary, these findings revealed the mechanism by which drying temperature modulates the sensory quality of ‘Qiancha 1’ white tea through the regulation of specific metabolites, such as the association between alkaloids and bitterness, organic acids and thickness, and terpenoids and aroma, thereby providing a scientific basis for the optimization of white tea processing.

### 3.4. Key Taste-Related Metabolite Analysis

**Figure 4:**
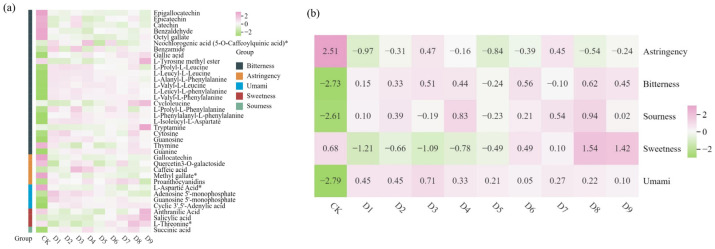
Effects of the key taste-related metabolites under different drying processes. (**a**) Heatmap of key taste-related metabolites. “*” indicates significance at the *p* < 0.05. (**b**) Heatmap of content levels by taste type for key taste-related metabolites. Note: The numbers and corresponding treatment conditions are as follows: D1 (65 °C drying for 1 h), D2 (65 °C drying for 2 h), D3 (65 °C drying for 3 h), D4 (75 °C drying for 1 h), D5 (75 °C drying for 2 h), D6 (75 °C drying for 3 h), D7 (90 °C drying for 1 h), D8 (90 °C drying for 2 h), and D9 (90 °C drying for 3 h). As shown in Figure 4b, the drying process reduced the content of astringent compounds but increased the levels of bitter, sour, and umami substances. Under drying at 65 °C, both astringent and bitter compounds showed an increasing trend with prolonged drying time. In contrast, the tea samples dried at 75 °C and 90 °C exhibited irregular changes with extended drying time, which might be attributed to interactive effects between temperature and duration. Sweetness-related metabolites accumulated progressively with longer drying time at 75 °C. Furthermore, elevated temperature and extended duration both contributed to the accumulation of sweet-tasting compounds. However, the E-tongue measurements indicated a decrease in sweet taste intensity under these conditions, possibly due to antagonistic interaction with bitter-tasting substances. The content of umami compounds gradually decreased with extended drying time at both 75 °C and 90 °C, and it also declined with increasing drying temperature, indicating that high temperature and long duration reduced umami intensity. This result was consistent with the E-tongue data. In conclusion, the unique taste profile of ‘Qiancha 1’ white tea was shaped by the combined effects of the types and concentrations of key taste-related metabolites, as influenced by specific drying conditions.

A total of 37 key taste-related metabolites were identified from 376 discrepant non-volatile metabolites through literature mining (Figure 4a). These included flavonoids (5), phenolic acids (9), amino acids and their derivatives (13), alkaloids (1), nucleotides (7), organic acids (1), and tannins (1). The five flavor-related flavonoids included quercetin3-O-galactoside, catechin, epicatechin, epigallocatechin, and gallocatechin. Among them, quercetin3-O-galactoside contributed to astringency, gallocatechin exhibited an astringent sensory profile, and catechin, epicatechin, and epigallocatechin were associated with bitterness [9,33]. Compared to CK, the contents of these flavonoids were significantly reduced in the samples of D1 to D9. This indicates that the drying process markedly diminishes the bitterness and astringency of white tea, thereby contributing to the sweet and mellow taste profile characteristic of ‘Qiancha 1’ white tea. The nine taste-related phenolic acids included sweetness-contributing compounds (anthranilic acid and salicylic acid), bitterness-associated compounds (benzaldehyde, octyl gallate, neochlorogenic acid (5-O-caffeoylquinic acid) *, benzamide, and gallic acid), and astringency-contributing compounds (caffeic acid and methyl gallate *) [33]. With an increase in drying time and temperature, the content of sweet and bitter phenolic acids increased, while astringent phenolic acids decreased in content. These changes were likely related to the key compounds underlying the formation of the sweet, mellow, and refreshing taste profile of ‘Qiancha 1’ white tea. The thirteen taste-related amino acids and their derivatives included bitter-tasting compounds (L-tyrosine methyl ester, L-prolyl-L-leucine, L-leucyl-L-leucine, L-alanyl-L-phenylalanine, L-valyl-L-leucine, L-leucyl-L-phenylalanine, L-valyl-L-phenylalanine, cycloleucine, L-prolyl-L-phenylalanine, L-phenylalanyl-L-phenylalanine, and L-isoleucyl-L-aspartate), sweet-tasting compound (L-threonine *), and umami-tasting compound (L-aspartic acid *) [33]. In the tea samples of D1 to D9, the contents of L-prolyl-L-leucine, L-leucyl-L-leucine, L-alanyl-L-phenylalanine, L-valyl-L-leucine, L-leucyl-L-phenylalanine, L-valyl-L-phenylalanine, L-prolyl-L-phenylalanine, and L-isoleucyl-L-aspartate showed a gradually decreasing trend, while the levels of L-tyrosine methyl ester, cycloleucine, and L-threonine* exhibited an increasing trend. The only taste-related alkaloid identified through screening was tryptamine, which was associated with a bitter taste profile [34]. Its content showed an increasing trend across the tea samples of D1 to D9, a finding consistent with the elevated bitterness intensity detected by the E-tongue in these samples. The only taste-related organic acid identified was succinic acid, which contributed sourness to the tea infusion. Similarly, the sole taste-active tannin compound was proanthocyanidins, which imparted astringency to the tea infusion. Among the seven taste-related nucleotides identified, adenosine 5’-monophosphate, guanosine 5’-monophosphate, and cyclic 3’,5’-adenylic acid imparted a distinct umami taste [8,35]. Their contents were consistently higher in the CK sample. This indicates that the drying process enhanced the umami characteristics of ‘Qiancha 1’ white tea. However, as drying temperature increased or duration extended, the contents of these umami-imparting nucleotides exhibited a decreasing trend, suggesting that specific combinations of drying parameters could further modulate umami intensity. Furthermore, cytosine, guanosine, thymine, and guanine are known as bitter-tasting compounds [33]. After drying, the contents of cytosine, guanosine, and guanine increased, while that of thymine decreased. Moreover, with increasing drying temperature or extended duration, the levels of cytosine and guanosine gradually declined, whereas guanine showed a consistent upward trend. These findings demonstrate that drying parameters significantly influence the bitterness profile of ‘Qiancha 1’ white tea.

### 3.5. Volatile Metabolite Analysis of ‘Qiancha 1’ White Tea

#### 3.5.1. Key Volatile Metabolite Analysis

**Figure 5:**
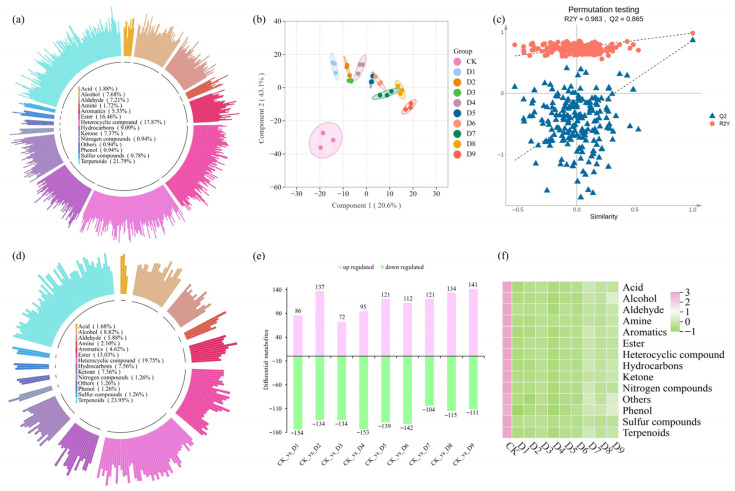
Volatile compound composition and multivariate analysis. (**a**) Number and percentages for each subcategory of all volatile compounds. (**b**) PLS-DA score plot. (**c**) Cross-validation results based on 200 calculations by using a permutation test. (**d**) Number and percentages for each subcategory of differential volatile compounds. (**e**) Mirror histogram of differential volatile compounds. (**f**) Differential volatile compound clustering heat map. Note: The numbers and corresponding treatment conditions are as follows: D1 (65 °C drying for 1 h), D2 (65 °C drying for 2 h), D3 (65 °C drying for 3 h), D4 (75 °C drying for 1 h), D5 (75 °C drying for 2 h), D6 (75 °C drying for 3 h), D7 (90 °C drying for 1 h), D8 (90 °C drying for 2 h), and D9 (90 °C drying for 3 h).

According to the GC-MS results (Table S7), a total of 638 volatile metabolites were detected in the tea samples, including the CK sample and the dried white tea samples (D1 to D9). They were categorized into 14 classes (Figure 5a), including 139 terpenoids (21.79%), 114 heterocyclic compounds (17.87%), 105 esters (16.46%), 58 hydrocarbons (9.09%), 49 alcohols (7.68%), 47 ketones (7.37%), 46 aldehydes (7.21%), 34 aromatic hydrocarbons (5.33%), 12 acids (1.88%), 11 amines (1.72%), 6 nitrogen-containing compounds (0.94%), 6 phenols (0.94%), 5 sulfur-containing compounds (0.78%), and 6 other compounds (0.94%).

To investigate the impact of different drying processes on the volatile metabolites of ‘Qiancha 1’ white tea, OPLS-DA was further employed to achieve group separation and calculate the VIP (variable importance in projection) value. Differential metabolites were selected based on the criteria of VIP > 1, Fold Change ≥ 2 or ≤0.5, and *p* < 0.05. The OPLS-DA score plot (Figure 5b) revealed clear separation among the 10 samples, with minimal within-group variation, indicating distinct alterations in volatile metabolite profiles resulting from different drying treatments and supporting the robustness of the data. A permutation test with 200 iterations was performed to validate the OPLS-DA model (Figure 5c). The intercepts of the regression lines for both the *Y*-axis and Q^2^ were below zero, demonstrating the absence of overfitting and confirming the reliability of the model [36]. A total of 238 differential volatile metabolites (DVMs) were identified in the 10 samples (Table S8). These metabolites were also categorized into 14 classes (Figure 5d), including 57 terpenoids (23.95%), 47 heterocyclic compounds (19.75%), 31 esters (13.03%), 21 alcohols (8.82%), 18 ketones (7.56%), 18 hydrocarbons (7.56%), 14 aldehydes (5.88%), 11 aromatic hydrocarbons (4.62%), 5 amines (2.10%), 4 acids (1.68%), 3 nitrogen-containing compounds (1.26%), 3 phenols (1.26%), 3 sulfur-containing compounds (1.26%), and 3 other compounds (1.26%). As shown in Figure 5f, the drying process significantly influenced the contents of DVMs, with changes following distinct patterns in response to increasing drying temperature and duration.

According to Figure 5f, the contents of volatile compounds were higher in CK than in the dried tea samples (D1 to D9), indicating that the overall content of volatile metabolites reduced due to the drying process. This was attributed to the evaporation of low-boiling-point volatile substances during drying. However, the total volatile content in the samples dried at a high temperature (90 °C) was higher than that in those dried at a low temperature (65 °C). This phenomenon might be explained by the increase in the content of terpenoids and the formation of pyrazines and pyrroles under high-temperature drying. This was consistent with the non-volatile metabolites results mentioned before. We observed that the contents of terpenoids, heterocyclic compounds, esters, hydrocarbons, alcohols, ketones, aldehydes, aromatic hydrocarbons, acids, amines, nitrogen-containing compounds, phenols, and sulfur-containing compounds were higher in the white tea sample dried at 90 °C compared to the other samples treated at other drying temperatures. This indicates that high-temperature (90 °C) drying promotes the formation of flavor compounds in ‘Qiancha 1’ white tea.

#### 3.5.2. ROAV Analysis for Key Volatile Metabolites

Relative odor activity values (rOAVs) are a method for identifying key flavor compounds in food by integrating the odor threshold of a compound. This method was used to evaluate the contribution of each aroma compound to the overall aroma profile of a sample. Generally, an rOAV ≥ 1 indicates that the compound directly contributes to the flavor of the sample, and a positive correlation is observed between the rOAV and the contribution of the corresponding compound to the flavor profile of the sample [37,38,39,40].

A total of 62 key volatile metabolites (KVMs) were identified from the 238 DVMs with rOAV ≥ 1 (Table S9). Among these KVMs, 14 compounds with rOAV ≥ 100 in the dried samples (D1 to D9) were selected for detailed analysis. They included 2-methoxy-3-(1-methylethyl)-pyrazine (rOAV = 227,578.83~700,854.26), 1-(2-thienyl)-ethanone (rOAV = 4132.61~19,445.29), tetrahydro-4-methyl-2-(2-methyl-1-propenyl)-2H-pyran (rOAV = 7152.99~17,162.49), linalool (rOAV = 3979.09~10,405.82), (E)-6-nonenal (rOAV = 3028.47~5919.72), (E,E)-3,5-octadien-2-one (rOAV = 1681.39~5435.78), 1-hexanol (rOAV = 226.34~2120.29), 2-methylisoborneol (rOAV = 1001.03~2004.01), p-cresol (rOAV = 668.18~1844.57), (Z)-4-heptenal (rOAV = 620.29~1167.69), 5-methyl-(E)-2-hepten-4-one (rOAV = 202.37~763.18), 3-mercaptohexyl acetate (rOAV = 259.57~495.58), methyl ester-benzoic acid (rOAV = 89.86~248.71), and nonanal (rOAV = 238.48~382.51). These compounds were considered to contribute significantly to the aroma characteristics of ‘Qiancha 1’ white tea. Notably, 2-methoxy-3-(1-methylethyl)-pyrazine had the highest rOAV of all the samples, indicating that it might be the most impactful volatile compound contributing to the distinctive aroma of ‘Qiancha 1’ white tea.

**Figure 6:**
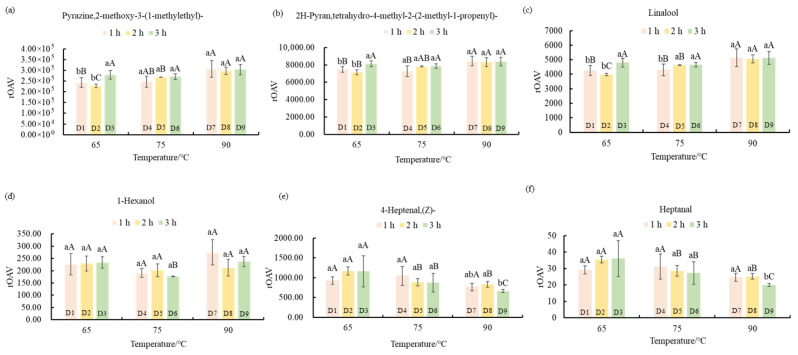
Effects of key volatile metabolites under different drying processes. Histogram of rOAV for 2-methoxy-3-(1-methylethyl)-pyrazine (**a**), tetrahydro-4-methyl-2-(2-methyl-1-propenyl)-2H-pyran (**b**), linalool (**c**), 1-hexanol (**d**), (Z)-4-heptenal (**e**), and heptanal (**f**). Note: The values are the mean of three repeated sensory ratings, and the error bars represent the standard error (*n* = 3). Lowercase letters represent the multiple comparison results of different drying times treated at the same temperature at the 0.05 level. Capital letters represent the multiple comparison results of the sensory evaluation scores of the tea samples. The same letter indicates no significant difference between the samples. The numbers and corresponding treatment conditions are as follows: D1 (65 °C drying for 1 h), D2 (65 °C drying for 2 h), D3 (65 °C drying for 3 h), D4 (75 °C drying for 1 h), D5 (75 °C drying for 2 h), D6 (75 °C drying for 3 h), D7 (90 °C drying for 1 h), D8 (90 °C drying for 2 h), and D9 (90 °C drying for 3 h).

2-methoxy-3-(1-methylethyl)-pyrazine imparted sweet, nutty, and caramel-like aroma attributes [10,34]. When drying for 2 h, its rOAV significantly increased with an increase in temperature (*p* < 0.05) (Figure 6a). Both extended drying time and elevated temperature effectively enhanced its content, explaining the pronounced caramel aroma observed in the ‘Qiancha 1’ white tea dried at 90 °C. Tetrahydro-4-methyl-2-(2-methyl-1-propenyl)-2H-pyran is characterized by sweet and floral notes [41]. It had the highest content in D3 (65 °C drying for 3 h) (Figure 6b). Its content also increased with increasing drying temperature. 1-hexanol, contributing fruity and sweet aromas [42], was not affected by drying time. However, its content was lowest when dried at 75 °C (Figure 6d). Linalool, known for its floral scent and ability to enhance the perception of sweetness in tea infusions [43,44], displayed the highest rOAV under 3 h drying across all temperatures. This indicates that prolonged drying time effectively increased its concentration. Furthermore, at a fixed drying duration of 2 h, the rOAV of linalool increased with rising temperature. This suggests that high drying temperatures promoted the accumulation of linalool. (E, E)-3,5-octadien-2-one and methyl ester benzoic acid have fruity and floral aromas, respectively [45]. This feature results in an increase in the perception of sweetness and fragrance [44]. Collectively, these compounds were key contributors to the characteristic sweet aroma profile of ‘Qiancha 1’ white tea.

Heptanal exhibits fresh and green aroma notes [43]. (Z)-4-heptenal is characterized by a green aroma [43]. When dried for 3 h, they both showed a significant decreasing trend in rOAV with increasing drying temperature (*p* < 0.05) (Figure 6f). Furthermore, at drying temperatures of 75 °C and 90 °C, their rOAV decreased with an extended drying time. These results indicate that both high drying temperatures and long drying times led to a reduction in the content of heptanal and (Z)-4-heptenal. The prolonged drying duration and elevated drying temperature were unfavorable for the retention of fresh and green aroma compounds. As the green aroma diminished due to extended drying time and increased temperature, the fresh aroma also decreased. This observation was consistent with the results of the sensory evaluation.

Drying plays a crucial role in the formation of the aroma profile of white tea [46,47,48]. At appropriate drying temperatures, protein denaturation terminated enzyme-mediated biochemical reactions, while low-boiling-point grassy compounds volatilized and high-boiling-point aroma substances were retained [49,50]. This mechanism was supported in the present study by the gradual decrease in the rOAV of heptanal and (Z)-4-heptenal with increasing drying temperature. Pyrazines are formed through the Maillard reaction, which occurs between sugars and amino acids under high-temperature conditions [9]. The observed decrease in the content of amino acids and their derivatives, alongside the increase in pyrazine-type aroma compounds with elevated drying temperature, confirmed the occurrence of the Maillard reaction during the drying process of ‘Qiancha 1’ white tea. During the drying process, varied physicochemical reactions driven by temperature and duration were key to the formation of the characteristic flavor profile of white tea.

## 4. Conclusions

This study systematically investigated the effects of different drying processes on the flavor quality of ‘Qiancha 1’ white tea through sensory evaluation, E-tongue analysis, and widely targeted metabolomics. The results demonstrated that drying temperature and duration significantly modulated the flavor characteristics of white tea. Low-temperature drying preserved a sweet, mellow, refreshing taste with a fresh and sweet aroma, while high-temperature drying imparted caramel and creamy notes at the expense of increased bitterness and reduced sweetness. A total of 37 key taste-active metabolites were identified, including bitter compounds (e.g., alkaloids), sweet compounds (e.g., phenolic acids), and umami compounds (e.g., nucleotides), whose dynamic changes directly influenced the taste profile of white tea. High-temperature drying (90 °C) significantly elevated the contents of volatile compounds, such as terpenoids, heterocyclics, and esters, particularly 2-methoxy-3-(1-methylethyl)-pyrazine, which exhibited the highest rOAV value and contributed distinct caramel and nutty aromas. In contrast, low-temperature and long-duration drying (65 °C, 3 h) preserved fresh aroma compounds (e.g., heptanal), while high-temperature drying accelerated their loss. To achieve a sweet, mellow, and refreshing flavor profile, the drying process at 65 °C for 3 h is recommended. If caramel notes and a rich aroma are desired, 90 °C drying may be applied, but the duration should be controlled to avoid excessive bitterness. Drying processes significantly shaped the flavor characteristics of ‘Qiancha 1’ white tea by regulating the accumulation and transformation of key metabolites. This study provided a theoretical foundation for the precise regulation of drying processes in white tea production, thereby enhancing the quality and market competitiveness of ‘Qiancha 1’ white tea.

## Data Availability

The original contributions presented in the study are included in the article/Supplementary Materials. Further inquiries can be directed to the corresponding authors.

## Supplementary Materials

The following supporting information can be downloaded at: https://www.mdpi.com/article/10.3390/foods14213787/s1, Table S1: Sensory evaluation results of ‘Qiancha 1’ white tea prepared by different drying processes; Table S2: ANOVA Tables for aroma and taste score variables; Table S3: Electronic tongue flavor intensity value of ‘Qiancha 1’ white tea prepared by different drying processes; Table S4: ANOVA Tables for electronic tongue flavor intensity value score variables; Table S5: List of the nonvolatile compounds in the white tea samples; Table S6: List of the discrepant nonvolatile compounds in the white tea samples; Table S7: List of the volatile compounds in the white tea samples; Table S8: List of the discrepant volatile compounds in the white tea sample; Table S9: rOAV values of key differential volatile compounds.
